# High Carriage Rate of Extended-Spectrum *β*-Lactamase-Producing *Escherichia coli* and *Klebsiella* Species among Poultry Meat Vendors in Dar es Salaam: The Urgent Need for Intervention to Prevent the Spread of Multidrug-Resistant Pathogens

**DOI:** 10.1155/2021/6653993

**Published:** 2021-03-03

**Authors:** Lutengano W. Mwanginde, Mtebe Majigo, Debora C. Kajeguka, Agricola Joachim

**Affiliations:** ^1^Department of Microbiology and Immunology, Muhimbili University of Health and Allied Sciences, P. O. Box 65001, Dar es Salaam, Tanzania; ^2^Directorate of Forensic Bureau, Ministry of Home Affairs, P. O. Box 9094, Dar es Salaam, Tanzania; ^3^Faculty of Medicine, Kilimanjaro Christian Medical University College, P. O. Box 2240, Moshi Kilimanjaro, Tanzania

## Abstract

**Background:**

Bacteria possessing extended-spectrum beta-lactamase (ESBL), especially *E. coli* and *Klebsiell*a species, are problematic, particularly in hospitalized patients. Poultry meat vendors are at risk of carrying ESBL-producing bacteria when processing and handling meat products in an unhygienic environment. There is limited information on the carriage rate of ESBL-producing pathogens among poultry meat vendors that necessitated the conduction of the study.

**Method:**

A cross-sectional study was conducted among poultry meat vendors in Dar es Salaam, Tanzania. Participants provided rectal swabs in transport media upon instruction. The primary isolation of ESBL-producing bacteria was carried out using MacConkey agar supplemented with ceftazidime. Identification of isolates relied on conventional methods. Double-disk synergy was the method used to confirm ESBL-producing isolates. We performed descriptive statistics using Statistical Package for Social Sciences version 23. A *p* value < 0.05 was considered statistically significant.

**Results:**

A total of 300 participants were recruited from five districts, with a mean age of 27.2 ± 6.7 years. The majority was male (67.3%), and 74.7% worked as poultry meat vendors for more than one year. Out of 300 participants, 107 (35.7%) had confirmed ESBL-producing *E. coli* and *Klebsiella* spp. The majority of confirmed ESBL-producing isolates was *E. coli* (78.5%). Participants from Ubungo District had significantly higher carriage of ESBL-producing *Escherichia coli* and *Klebsiella* spp. (48.0%, 95% CI: 34.8–47.7) than Temeke District (21.4%, 95% CI: 13.4–32.4). Only 28.0% of participants had access to latrines at the workplace, and all working areas lacked access to running water.

**Conclusion:**

The study revealed a relatively high fecal carriage rate of ESBL-producing *E. coli* and *Klebsiella spp*. among poultry meat vendors. Poor working environments and hygienic practices are risks for spread of these *multidrug-resitant* pathogens.

## 1. Introduction

Extended-spectrum *β*-lactamase- (ESBL-) producing pathogens are among the common causes of bacterial infections with increased antibiotic resistance [1]. Infection due to ESBL-producing pathogens is associated with high morbidity and mortality [2, 3]. There is evidence of growing antimicrobial resistance in animals related to varying ESBL alleles [4, 5]. Of concern is the spread of ESBL-producing pathogens to humans through food substances, particularly animal food products [6, 7].

The irrational use of antimicrobial agents in the animal influence the emergency of antibiotic-resistant strains [8, 9] and subsequently spread to humans through consumption of animal products contaminated with resistant stains [10, 11]. The use of antibiotics in poultry is a common practice in several countries [12–14]. Hence, poultry meat may serve as a source of spread of ESBL strains to humans [15–17]. In particular, studies have shown that more than 90% of chicken meat carry ESBL-producing pathogens [16, 18]. In this regard, poultry meat vendors have a high chance of contact with ESBL-producing pathogens [19]. Poultry meat vendors expose during slaughtering, preparation, and selling of meat; considerably, their working environments in low-income countries are relatively not clean and unsafe [20]. The association between poultry meat and human health is becoming an essential issue in the poultry industry versus food safety in the fight against antimicrobial resistance [21].

Most studies in Tanzania on the carriage rate of ESBL-producing pathogens in humans were conducted in hospital settings [22, 23]. *E. coli* and *K. pneumoniae* are reported in these studies as the common ESBL-producing pathogens [22, 23]. On the contrary, the ESBL carriage rate among poultry meat vendors in the community has not been comprehensively studied. The current study investigated the fecal carriage rate of ESBL-producing *E. coli* and *Klebsiella* spp. among the poultry meat vendors in Dar es Salaam, Tanzania. The findings provide baseline information to guide strategies for the control of infection and antimicrobial resistance in community settings.

## 2. Materials and Methods

### 2.1. Study Design, Setting, and Population

This was a cross-sectional study conducted in Dar es Salaam, the largest city in Tanzania. The study involved poultry slaughterhouses and poultry meat-selling centers from selected wards in five districts of the Dar es Salaam region. The study recruited healthy poultry meat vendors involved in either poultry slaughtering, washing, or selling raw meat for more than three months. The study excluded vendors with signs of illness and those who failed to provide written informed consent.

### 2.2. Sample Size and Sampling Procedure

We determined the sample size using the Kish Leslie formula [24], considering a 24.3% prevalence of ESBL in Dar es Salaam [25], 95% confidence interval, and 5% margin of error. The minimum sample size required for the study was 283. Our approach to the selection of wards and participants in five districts used a multistage sampling technique. The wards with poultry houses and meat-selling centers per district were obtained. After that, the total number of wards from five districts was used as the denominator to calculate the sample size to be drawn per each district. We based on a proportion to size sampling technique to obtain the number of wards per district to get 23 out of 107 wards in Dar es Salaam. Two to seven wards per district and about 10 to 20 participants from each ward were recruited by simple random sampling at the poultry meat vendor centers.

### 2.3. Data Collection

The investigator visited poultry meat vendors at their working station for data collection. The interviewer provided a detailed explanation about the study to every study participant who provided written informed consent before recruitment. The data were collected using a structured questionnaire. The collected information includes sociodemographic characteristics (age, sex, location, and duration in business), working environments, and hygienic practice conditions.

### 2.4. Sample Collection and Transportation

The research assistant clearly instructed each participant and illustrated the rectal swab's self-collection and how to insert the swab onto Cary-Blair transport media. The samples were transported daily in a cool box with ice packs within six hours after collection to the microbiology and immunology laboratory at Muhimbili University of Health and Allied Sciences (MUHAS) for processing.

### 2.5. Laboratory Procedure

Each swab was inoculated on MacConkey agar, supplemented with 1.0 mg/L ceftazidime, within 24 hours after collection. The inoculated plates were incubated aerobically at 37°C for 18 to 24 hours. The presence of ceftazidime in the media inhibits the growth of non-ESBL-producing bacteria; therefore, isolated colonies were presumptively considered as ESBL-producing pathogens. We first suspected *E. coli* and *Klebsiella* spp. based on colonial characteristics and Gram stain reaction. Colonies with distinctive morphological appearance of dry or mucoid lactose-positive (pink) and Gram-negative rods were selected. Suspected colonies were subcultured into nutrient agar (Oxoid Ltd., UK) to get enough pure colonies for further identification. Conventional biochemical tests of Kligler's iron agar, sulfide-indole-motility test, and citrate test were used (Table 1).

**Figure 1:**
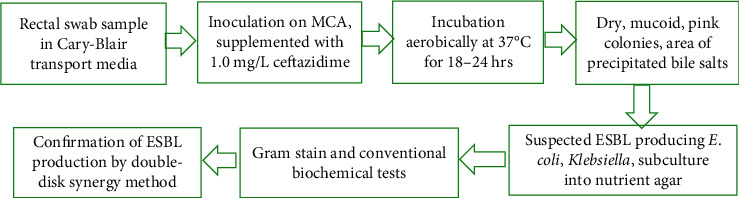
Laboratory workflow for isolation, identification, and confirmation of ESBL-producing *E. coli* and *Klebsiella* spp.

As previously described, it was confirmed that all isolates were ESBL-producing pathogens by the double-disk synergy method [26]. In brief, Muller Hinton agar plates were inoculated with suspension of isolates matching 0.5 McFarland turbidity standards. Ceftazidime (30 *μ*g) and cefotaxime (30 *μ*g) disks were placed at a distance of 20 mm around the amoxicillin-clavulanic acid (30 *μ*g) disk. The incubation environment for inoculated media was 37°C for 18 to 24 hours. Any distortion or increase in the inhibition zone towards the disk of amoxicillin-clavulanic acid indicated positive for ESBL production. *Klebsiella pneumoniae* ATCC 700603 represented a positive control strain for ESBL production. The laboratory workflow for isolation, identification, and confirmation of ESBL-producing *E. coli* and *Klebsiella* spp. is summarized in Figure 1.

### 2.6. Data Analysis

Data were analyzed using Statistical Package for Social Sciences version 23.0. Categorical variables were summarized as proportions, while continuous variables were summarized as mean and standard deviation. A chi-square test was calculated to determine the differences between proportions. A 95% confidence interval of proportion was calculated to provide more information on the upper and lower bound of proportion estimates. The level of significance was specified at 0.05.

## 3. Results

### 3.1. Demographic Characteristics of Study Participants

The study enrolled 300 poultry meat vendors with a mean age of 27.2 ± 6.7 years. The majority, 202 (67.3%), was males, and 138 (46.0%) were below 25 years. More than a quarter, 80 (26.7%), were recruited from the Kinondoni District, followed by Temeke District 70 (23.3%). Of all, 224 (74.7%) worked as poultry meat vendors for more than one year. Around half, 151 (50.3%), reported a preference for self-medication with antibiotics before going to the hospital (Table 2).

### 3.2. Working Environment and Hygiene Practice

Most of the study participants, 216 (72%), had no latrines at their workplace, and all (100%) reported having access to water for washing hands, clearing the environment, cleaning knives, washing poultry, bowel, and plates. However, all working areas lack access to running water. No participant used hand protection when handling poultry meat (data not presented).

### 3.3. Distribution of ESBL Isolates

A total of 130 (43.3%) bacterial isolates were suspected to be ESBL-producing *E. coli* and *Klebsiella* spp. *E. coli*, 101 (77.7%), were the predominant bacteria. Of 130 ESBL-suspected isolates, 107 (82.3%) were confirmed as ESBL-producing *E. coli* and *Klebsiella* spp. Out of 107 ESBL producers, 84 (78.5%) were *E. coli* isolates, and 23 (21.5%) were *Klebsiella* spp. (Table 3).

### 3.4. The Proportion of ESBL-Producing Bacteria among Study Participants

The overall carriage rate of ESBL-producing *E. coli* and *Klebsiella* spp. was 35.7% (95% CI: 30.5–41.2). The frequency of carriage was high (38.4%) among participants aged less than 25 years, but the observed difference in age groups was not significant. There was a significant difference in the proportion of participants with carriage of ESBL-producing *E. coli* and *Klebsiella* spp. in the district of business. Ubungo District had higher proportion of fecal carriage (48.0%, 95% CI: 34.8–47.7) than Temeke District (21.4%, 95% CI: 13.4–32.4) (*p*=0.030). The proportion of fecal carriage of ESBL-producing *E. coli* and *Klebsiella* spp. observed in sex, duration in business, and preference for self-treatment was not significantly different (*p* > 0.05) (Table 4).

## 4. Discussion

The present study demonstrates a high carriage rate of ESBL-producing *E. coli* and *Klebsiella* spp. among poultry meat vendors in Dar es Salaam. Although there is no study done in a similar population in Tanzania, the finding is relatively higher than the fecal carriage of ESBL-producing bacteria in the community among people sharing latrines in Dar es Salaam [25]. The study also revealed poor working environment and hygienic practices in the poultry meat vendor. Based on these findings, the high fecal carriage of ESBL-producing *E. coli* and *Klebsiella* spp. may be due to direct contact with poultry meat that may be carrying or contaminated with ESBL strains, poor hygiene practices in their working environment, and lack of safe and running water for washing hands and other uses.

In the current study, *E. coli* was the most common ESBL producer isolated among poultry meat vendors than *Klebsiella* spp.; the finding agrees with the study reported in Spain [27]. The predominance of *E. coli* was also reported in another study conducted in Tanzania, where *E. coli* was the most common cause of community-acquired infection [28]. Furthermore, a study done in Mwanza on companion and domestic animal carriage of ESBL-producing bacteria reported the predominance of *E. coli* at a rate of 21.7% [5]. The study from Gabon, sub-Saharan Africa, reported a 23.0% rate of chicken contamination with ESBL-producing *E. coli* [29]. Thus, the predominance of ESBL-producing *E. coli* among poultry meat vendors indicates a community problem as these poultry meat vendors are part of the community.

Our study revealed the lack of running water in the working area for all poultry meat vendors. Instead, they used water kept in the buckets or tins for washing hands, poultry meat, and cleaning tables. Hand washing from the same bucket may increase the contamination rate, thus spreading the ESBL-producing bacteria. Studies in France reported the prevalence of 10.7% of ESBL-producing *E. coli* in humans related to contamination during slaughtering and in contact with food animal products, including poultry meat [30]. A Dutch study reported an 18% contribution of poultry to ESBL-producing *E. coli* exposure of the Dutch population through chicken meat consumption [31]. Compared to our findings, the difference in prevalence could be contributed by the level of personal hygienic practices [30]. Handling contaminated animal food products increases the risk of transmission of ESBL bacteria [15, 20]. In this study, all poultry meat vendors did not use hand protective gear such as gloves when processing poultry meat and serving customers, hence directly contacted poultry meat. The study enrolled participants from five districts in Dar es Salaam; the fecal carriage rate of ESBL showed significant difference between some districts. The prevalence between Ubungo and Temeke Districts cannot be explained with available data for demographic and hygienic practice. The findings call for a comprehensive and detailed comparative study on the demographic characteristics, working environment, and hygienic practice.

To the best of our knowledge, this is the first report from the Dar es Salaam region investigating the prevalence of ESBL-producing bacteria in poultry meat vendors. Therefore, our study presents the baseline information on the prevalence of ESBL-producing *E. coli* and *Klebsiella* spp. among poultry meat vendors. The present study's findings suggest the need for a regular screen for colonization with ESBL-producing bacteria for a specific group of poultry meat vendors and improving the working environment and hygienic practices. The government should have a policy that requires the screening of food handlers before engaging in business to control the spread of ESBL-producing bacteria and antimicrobial resistance in the community. Based on the laboratory methods used to identify *E. coli and Klebsiella* spp., we might have underestimated these organisms' magnitude. Some strains may have an opposite reaction with the conventional biochemical tests used for identification, which advanced identification technique.

## 5. Conclusion

The study demonstrates a relatively high fecal carriage rate of ESBL-producing *E. coli* and *Klebsiella* spp. among poultry meat vendors. For food handlers, the availability of clean running water is essential as washing hands in the same water as where the chickens are washed is a risk for public health as well as for the poultry workers.

## Figures and Tables

**Table 1:** Biochemical characteristics used for identification of *E. coli and Klebsiella* spp.

Isolate	KIA	H_2_S	Indole	Motility	Citrate test
*E. coli*	A/A	−	+	+	−
*Klebsiella* spp.	A/A	−	−	−	+

KIA: Kligler's iron agar; H_2_S: hydrogen sulfide; A/A: acid/acid; (−): negative reaction; (+): positive reaction.

**Table 2:** Demographic distribution of study participants.

Variables	Frequency/mean ± SD	Percentage (%)
Mean age (years)	27.2 ± 6.7	
Age group (years)		
<25	138	46.0
25–30	85	28.3
>30	77	25.6
Sex		
Male	202	67.3
Female	98	32.7
Districts of business		
Ubungo	50	16.7
Ilala	60	20.0
Temeke	70	23.3
Kigamboni	40	13.3
Kinondoni	80	26.7
Duration in the business		
Three months to one year	76	25.3
More than one year	224	74.7
Preference for self-medication		
Yes	151	50.3
No	149	49.7

SD: standard deviation.

**Table 3:** Distribution of ESBL-producing *Escherichia coli* and *Klebsiella* species.

	Frequency	Percentage (%)
Primary ESBL isolation (*n* = 130)		
*E. coli*	101	77.7
*Klebsiella* spp.	29	22.3
ESBL confirmation (*n* = 107)		
*E. coli*	84	78.5
*Klebsiella* spp.	23	21.5

**Table 4:** Proportion of confirmed ESBL producers in study participants.

Variable	Total population	Confirmed ESBL producer		^*∗*^ *p* value
		*N*	% (95% CI)	
Overall	300	107	35.7 (30.5–41.2)	
Age group (years)				0.565
<25	138	53	38.4 (30.7–46.7)	
25–30	85	26	30.6 (21.8–41.1)	
>30	77	28	36.4 (26.5–47.5)	
Sex				0.599
Male	202	70	34.7 (28.4–41.4	
Female	98	37	37.8 (28.8–47.7)	
Districts of business				0.030
Ubungo	50	24	48.0 (34.8–61.5)	
Ilala	60	25	41.7 (30.1–54.3)	
Temeke	70	15	21.4 (13.4–32.4)	
Kigamboni	40	13	32.5 (20.1–48.0)	
Kinondoni	80	30	37.5 (27.7–48.5)	
Duration in business				0.804
≥3months to 1 year	76	28	36.8 (26.9–48.1)	
>1 year	224	79	32.4 (29.3–41.7)	
Preference for self-medication			0.836	
Yes	151	53	35.1 (27.9–43.0)	
No	149	54	36.2 (27.0–44.2)	

^*∗*^ *p* value according to Pearson's chi-square test. CI = confidence interval.

## Data Availability

All the relevant data generated and analyzed during this study are included within this manuscript. However, the dataset can be accessed from the corresponding author upon reasonable request.

## Abbreviations

ESBL: Extended-spectrum beta-lactamase
KIA: Kligler's iron agar
MUHAS: Muhimbili University of Health and Allied Sciences.
